# Drug hypersensitivity syndrome induced by sulfasalazine: A case report

**DOI:** 10.1097/MD.0000000000030060

**Published:** 2022-08-19

**Authors:** Dong-Hui Chen, Hai-Rong Zhou, Yong-Gang Zhang, Guan-Yuan Shen, Chong Xu, Chun-Li Guan

**Affiliations:** a Department of General Medicine, Shenzhen Longhua District Central Hospital, Shenzhen, China; b Department of Clinical Laboratory, Shenzhen Longhua District Central Hospital, Shenzhen, China.

**Keywords:** drug hypersensitivity syndrome, sulfasalazine

## Abstract

**Introduction:**

Drug hypersensitivity syndrome (DHS) induced by sulfasalazine is a serious systemic delayed adverse drug reaction, which is associated with significant morbidity and mortality.

**Patient concerns:**

A 52-year-old man was hospitalized for developing a rash after 3 weeks of sulfasalazine treatment for ulcerative colitis (UC).

**Diagnosis:**

The patient was diagnosed with DHS based on his drug history, clinical manifestations, and laboratory test results.

**Interventions:**

The patient was administered intravenous glucocorticoids. The patient’s condition improved after treatment with human immunoglobulin and antihistamines.

**Outcomes:**

Combination therapy of glucocorticoid and gamma globulin, the whole-body pruritus disappeared, and no new rash appeared. The whole-body rash subsided or turned dark red.

**Conclusion:**

This article describes the diagnosis and treatment process of a case of sulfasalazine-induced DHS and reviews the relevant literature to improve clinician understanding and avoid misdiagnosis and missed diagnosis.

## 1. Introduction

Sulfasalazine is metabolized to sulphapyridine and 5-aminosalicylic acid, which are widely used in the treatment of various autoimmune diseases. Drug hypersensitivity syndrome (DHS) induced by sulfasalazine is a serious systemic delayed adverse drug reaction that typically manifests at 2 to 6 weeks after drug initiation and can be fatal.^[1,2]^ It features many adverse reactions, including fever, rash, sore throat, muscle soreness, lymphadenopathy, blood dyscrasias, and hepatitis.^[3,4]^

Despite the significant amount of data on sulfasalazine-induced hypersensitivity syndrome, the diversity of its treatments has not been well described. Here we report the case of a patient with previously unidentified sulfasalazine-induced hypersensitivity syndrome who was not sensitive to high-dose hormone therapy alone.

## 2. Ethics and Methods

Written informed consent was obtained from the institutional medical ethics committee of Shenzhen Longhua Central Hospital. All procedures described in this case report involving the patient were performed in accordance with the 1964 Helsinki Declaration and its later amendments or comparable ethical standards.

## 3. Case presentation

**Figure 1.**
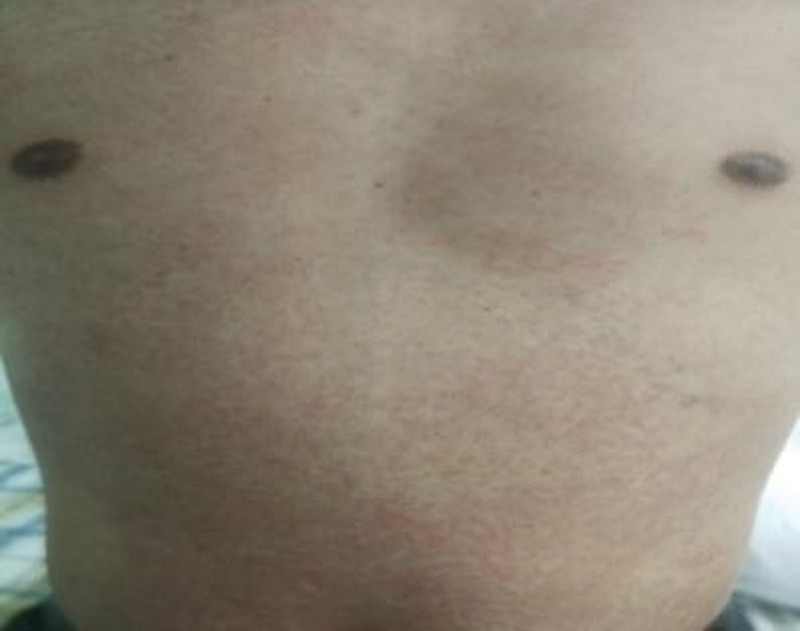
Red rash papules on the trunk.

**Figure 2.**
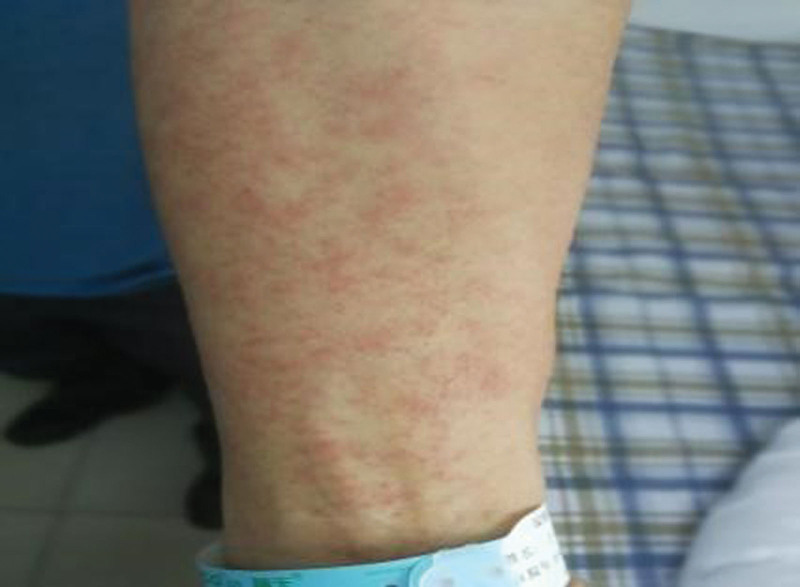
Scarlet rash papules on the limbs.

The patient, a 52-year-old man, was hospitalized on November 19, 2021 with systemic erythema and papules for 10 days and fever for 2 days. The patient underwent a colonoscopy on October 18, 2021, was diagnosed with ulcerative colitis (UC), and was started on sulfasalazine. After 3 weeks of treatment, a rash developed. The first scattered red maculopapular rash appeared on his face. The sulfasalazine was discontinued, but the rash did not decrease; rather, it gradually expanded to the trunk and limbs (Figs. 1 and 2). On March 18, he developed a fever with a maximum body temperature of 39.1°C. After loratadine administration, the rash did not decrease, and he gradually developed facial swelling and pain in the preauricular, neck, and groin areas. He denied a history of infectious diseases such as hepatitis and tuberculosis and had no history of food or drug allergies. He was admitted to the hospital with a pulse of 108 beats/min, blood pressure of 120/92 mm Hg, scattered erythema, papules, and pruritus throughout the body. Multiple enlarged lymph nodes were palpable in the bilateral preauricular, anterior neck, and inguinal regions with a soft texture, smooth surface, positive tenderness, and no palpable liver or spleen under the ribs. A cardiopulmonary abdominal examination showed no abnormalities. Laboratory examinations revealed negative results for high-sensitivity C-reactive protein (14 mg/L), anti-O antibody, and anti-nuclear antibody spectrum. Routine blood test results were as follows: white blood cell count, 14.8 × 10^9^/L; lymphocytes, 5.15 × 10^9^/L; neutrophils, 7.86 × 10^9^/L; monocytes, 0.99 × 10^9^/L; eosinophils, 0.7 × 10^9^/L; basophils, 0.09 × 10^9^/L; and alanine aminotransferase, 76 U/L. Color Doppler ultrasonography was used to examine the lymph nodes of the bilateral neck, supraclavicular, and groin with a largest diameter of 2.6 cm.

After hospitalization, intravenous methylprednisolone 80 mg, orally disintegrating loratadine tablet 10 mg at night, glycyrrhizin capsules 150 mg 3 times a day, and hydrocortisone butyrate topical was administered. After 3 days, the rash did not subside; rather, it gradually increased. On March 21, gamma globulin 10 g/d was added to the treatment plan, and no new rash appeared for 2 consecutive days. On March 23, the gamma globulin was increased to 25 g/d for 5 consecutive days, and the color of the patient’s whole-body rash gradually darkened and subsided, while his swollen lymph nodes gradually subsided. After discontinuation of the gamma globulin therapy, the patient continued taking methylprednisolone, which was gradually reduced to 28 mg orally for 5 days. He was then discharged from the hospital on oral methylprednisolone 12 mg. After discharge, the whole-body pruritus disappeared, and no new rash appeared. The whole-body rash subsided or turned dark red.

## 4. Discussion

Sulfasalazine is widely used in the treatment of UC,^[5]^ and clinicians should pay special attention to its possible serious systemic adverse reactions, of which 10% of DHS cases can be fatal.^[6,7]^ DHS is a rare adverse reaction that can be life-threatening in severe cases. One of its important features is a long incubation period, and symptoms usually appear 2 to 6 weeks after sensitization.^[8–10]^ The early rash manifests as scattered red macules and maculopapular rashes that usually first appears on the face and then extended to the trunk and limbs. The rash gradually increases from top to bottom. Patients often present with itchy skin, fever, facial swelling, and generalized lymphadenopathy. The rash first appears on the distal limbs and then on the trunk and face. Unlike general drug eruptions, the rash does not subside quickly after discontinuation of the sensitizing drug, and patients may require further care when hormone therapy is discontinued prematurely.^[11–13]^

The exact pathogenesis of DHS is currently unclear, but it is generally believed to be mediated by CD8+ T cells.^[14]^ Genetic susceptibility, immune status, reactivation of latently infected virus, and other factors are related, and how these factors interact is currently unclear.^[8,15,16]^ However, uniformly recognized worldwide diagnostic criteria are lacking. The diagnostic criteria in Europe and Japan are widely used in clinical practice,^[17–19]^ that is, DHS can be diagnosed according to the characteristics of the Chinese population as follows: delayed-onset rash, more than 3 weeks from medication to rash; swollen lymph nodes at 2 or more sites; body temperature >38°C; visceral damage: alanine transaminase level more than twice the normal value, interstitial nephritis, interstitial pneumonia, or myocarditis; hematological abnormalities including increased or decreased white blood cell count, eosinophil count ≥1.5 × 10^9^/L, or atypical lymphocytes >5%; and recurrence: although the drugs were discontinued and treatment was administered, the disease relapsed or became aggravated. The first 5 criteria can confirm the diagnosis of DHS.^[10]^

Glucocorticoids and gamma globulin are the main drugs used to treat DHS. In recent years, early hormone use has been recommended, generally methylprednisolone 1.0 to 1.5 mg/kg/d, the dose of which was gradually reduced after disease control was achieved. Because the pathogenesis of the disease is related to drug excretion, immunity, and reactivation of latent viral infection, the time for the final use of hormones differs widely. It is often necessary to use glucocorticoids for 5 to 10 weeks, and to prematurely stop glucose. Corticosteroid use can also lead to relapse. In recent years, immunoglobulin has been widely used for the treatment of severe drug eruptions. The general dosage was 0.2 to 0.4 g/kg/d for 3 to 5 days. If the effect is not evident, the dosage can be increased to 0.6 to 0.8 g/kg/d. Immunoglobulin can significantly relieve symptoms, reduce the risk of infection, reduce the glucocorticoid dosage, and improve rescue and cure rates.^[7,10,12,20]^ Therefore, the early use of glucocorticoids and high-dose gamma globulin can reduce patient mortality rates.

## Acknowledgments

We thank the patient, who agreed to the publication of his images and clinical information.

## Author contributions

Dong-Hui Chen: Design of the study; acquisition and interpretation of data; manuscript preparation and the initial draft; accountable for all aspects of the work. Yong-Gang Zhang: Accountable for all aspects of the work. Zhou Hai-Rong and Guan-Yuan Shen: analysis and interpretation of data; accountable for all aspects of the work. Chun-Li Guan and Chong Xu: design of the study; critical review of the draft and contribution to the writing of the manuscript; final approval of the version to be published and accountable to the accuracy or integrity of the work.

## References

[1] Mennicke M Zawodniak A Keller M. Fulminant liver failure after vancomycin in a sulfasalazine-induced DRESS syndrome: fatal recurrence after liver transplantation. Am J Transplant. 2009;9:2197–202. 19706026 10.1111/j.1600-6143.2009.02788.x

[2] Ribe J Benkov KJ Thung SN. Fatal massive hepatic necrosis: a probable hypersensitivity reaction to sulfasalazine. Am J Gastroenterol. 1986;81:205–8. 2869683

[3] Poland GA Love KR. Marked atypical lymphocytosis, hepatitis, and skin rash in sulfasalazine drug allergy. Am J Med. 1986;81:707–8. 2876632 10.1016/0002-9343(86)90562-0

[4] Brooks H Taylor HG Nichol FE. The three week sulphasalazine syndrome. Clin Rheumatol. 1992;11:566–8. 1362531 10.1007/BF02283121

[5] Chinese consensus on diagnosis and treatment in inflammatory bowel disease (2018, Beijing). J Dig Dis. 2021;22:298–317. 33905603 10.1111/1751-2980.12994

[6] Miyagawa F Asada H. Current perspective regarding the immunopathogenesis of drug-induced hypersensitivity syndrome/drug reaction with eosinophilia and systemic symptoms (DIHS/DRESS). Int J Mol Sci . 2021;22:2147. 33670052 10.3390/ijms22042147 PMC7927043

[7] Wu X Yang F Chen S. Clinical, viral and genetic characteristics of drug reaction with eosinophilia and systemic symptoms (DRESS) in Shanghai, China. Acta Derm Venereol. 2018;98:401–5. 29242946 10.2340/00015555-2867

[8] Musette P Janela B. New insights into drug reaction with eosinophilia and systemic symptoms pathophysiology. Front Med (Lausanne). 2017;4:179–179. 29255708 10.3389/fmed.2017.00179 PMC5722807

[9] Shiohara T Kano Y Takahashi R. Drug-induced hypersensitivity syndrome: recent advances in the diagnosis, pathogenesis and management. Progress Allergy. 2012;97:122–38. 10.1159/000335624 22613858

[10] Tohyama M. Drug-induced hypersensitivity syndrome. Nippon Rinsho. Japanese J Clin Med. 2012;70:498–502. 23156558

[11] Ben M’ rad M rad M Leclerc-Mercier S. Drug-induced hypersensitivity syndrome: clinical and biologic disease patterns in 24 patients. Medicine (Baltim). 2009;88:131–40. 10.1097/MD.0b013e3181a4d1a1 19440116

[12] Chen YC Cho YT Chang CY. Drug reaction with eosinophilia and systemic symptoms: a drug-induced hypersensitivity syndrome with variable clinical features. Dermatologica Sinica. 2013;31:196–204.

[13] Avancini J Maragno L Santi CG. Drug reaction with eosinophilia and systemic symptoms/drug-induced hypersensitivity syndrome: clinical features of 27 patients. Clin Exp Dermatol. 2015;40:851–9. 26271788 10.1111/ced.12682

[14] Niu J Jia Q Ni Q. Association of CD8(+) T lymphocyte repertoire spreading with the severity of DRESS syndrome. Sci Rep. 2015;5:9913–9913. 25905582 10.1038/srep09913 PMC4649994

[15] Drago F Cogorno L Broccolo F. A fatal case of DRESS induced by strontium ranelate associated with HHV-7 reactivation. Osteoporos Int. 2016;27:1261–4. 26519419 10.1007/s00198-015-3384-7

[16] Cheng CY Su SC Chen CH. HLA associations and clinical implications in T-cell mediated drug hypersensitivity reactions: an updated review. J Immunol Res. 2014;2014:565320. 24901010 10.1155/2014/565320 PMC4034438

[17] Shiohara T Iijima M Ikezawa Z. The diagnosis of a DRESS syndrome has been sufficiently established on the basis of typical clinical features and viral reactivations. Br J Dermatol. 2007;156:1083–4. 17381452 10.1111/j.1365-2133.2007.07807.x

[18] Kim DH Koh YI. Comparison of diagnostic criteria and determination of prognostic factors for drug reaction with eosinophilia and systemic symptoms syndrome. Allergy Asthma Immunol Res. 2014;6:216–21. 24843796 10.4168/aair.2014.6.3.216 PMC4021239

[19] Kardaun SH Sidoroff A Valeyrie-Allanore L. Variability in the clinical pattern of cutaneous side-effects of drugs with systemic symptoms: does a DRESS syndrome really exist. Br J Dermatol. 2007;156:609–11. 17300272 10.1111/j.1365-2133.2006.07704.x

[20] Chen X Wang S Li L. A case of drug-induced hypersensitivity syndrome induced by icotinib managed by intravenous immunoglobulin and systemic corticosteroids. Indian J Dermatol Venereol Leprol. 2018;84:350–2. 29516901 10.4103/ijdvl.IJDVL_490_17

